# Stochasticity in Natural Forage Production Affects Use of Urban Areas by Black Bears: Implications to Management of Human-Bear Conflicts

**DOI:** 10.1371/journal.pone.0085122

**Published:** 2014-01-08

**Authors:** Sharon Baruch-Mordo, Kenneth R. Wilson, David L. Lewis, John Broderick, Julie S. Mao, Stewart W. Breck

**Affiliations:** 1 Department of Fish, Wildlife, and Conservation Biology and Graduate Degree Program in Ecology, Colorado State University, Fort Collins, Colorado, United States of America; 2 Department of Fish, Wildlife, and Conservation Biology, Colorado State University, Fort Collins, Colorado, United States of America; 3 Terrestrial Programs, Colorado Parks and Wildlife, Fort Collins, Colorado, United States of America; 4 Terrestrial Programs, Colorado Parks and Wildlife, Glenwood Springs, Colorado, United States of America; 5 United States Department of Agriculture, Animal and Plant Health Inspection Service, Wildlife Services, National Wildlife Research Center, Fort Collins, Colorado, United States of America; University of Sydney, Australia

## Abstract

The rapid expansion of global urban development is increasing opportunities for wildlife to forage and become dependent on anthropogenic resources. Wildlife using urban areas are often perceived dichotomously as urban or not, with some individuals removed in the belief that dependency on anthropogenic resources is irreversible and can lead to increased human-wildlife conflict. For American black bears (*Ursus americanus*), little is known about the degree of bear urbanization and its ecological mechanisms to guide the management of human-bear conflicts. Using 6 years of GPS location and activity data from bears in Aspen, Colorado, USA, we evaluated the degree of bear urbanization and the factors that best explained its variations. We estimated space use, activity patterns, survival, and reproduction and modeled their relationship with ecological covariates related to bear characteristics and natural food availability. Space use and activity patterns were dependent on natural food availability (good or poor food years), where bears used higher human density areas and became more nocturnal in poor food years. Patterns were reversible, i.e., individuals using urban areas in poor food years used wildland areas in subsequent good food years. While reproductive output was similar across years, survival was lower in poor food years when bears used urban areas to a greater extent. Our findings suggest that bear use of urban areas is reversible and fluctuates with the availability of natural food resources, and that removal of urban individuals in times of food failures has the potential to negatively affect bear populations. Given that under current predictions urbanization is expected to increase by 11% across American black bear range, and that natural food failure years are expected to increase in frequency with global climate change, alternative methods of reducing urban human-bear conflict are required if the goal is to prevent urban areas from becoming population sinks.

## Introduction

A milestone was reached in 2008 when more than half of the world's population resided in urban areas; by 2050, 70% of the world's population will consist of urban residents, with more than half expected to live in small urban centers [1]. Ecological effects of urbanization are long-lasting and include land transformations, biotic modifications, and changes to biogeochemical cycles [2]–[4]. Furthermore, urbanization can affect individual wildlife and populations either negatively or positively, where negative effects include increased human-related stress, reduced forage quality, and reduced survival and reproductive success, and where positive effects include reduced predation pressure, increased availability of resources such as food and cover, and increased survival and reproduction success [5]–[9]. The latter positive effects can result in exploitation of, and reliance on, anthropogenic resources by wildlife, which can result in property damage, risks to human safety, and overall human-wildlife conflict [10]–[12].

Urban areas offer novel environments with spatially concentrated, highly productive, and temporally predictable resources [7], [13]–[14]. Wildlife using urban areas often possess behavioral traits that allow exploitation of such novel environments including generalized diets, high learning capacity, and behavioral plasticity (e.g., [9], [15]–[16]), and when individuals apply these traits to use anthropogenic resources, behavioral changes can ensue. Urban wildlife presumably need less area to obtain adequate resources compared to their wildland counterparts, and they may exploit resources during times that allow avoidance of high human activity. Evidence across taxa concurs, with urban individuals having smaller territories and home range sizes (e.g., [14], [17]–[18]) and modifying their normal activity patterns (e.g., [19]–[21]).

Bears are omnivores, have high learning capacity, and exhibit behavioral plasticity [22]–[23], traits that make them successful in exploiting anthropogenic resources in urban areas. Bears enter a state of intense feeding, or hyperphagia, during late summer and fall to gain energy reserves for winter hibernation [24]. During hyperphagia, bears subsist mainly on plant species that produce hard- and soft-mast, and in years of mast failure, they can move extensively in search of food and may forage on alternative anthropogenic sources near human development [25]–[27]. When bears use anthropogenic resources they can exhibit behavioral changes including having smaller home ranges compared to wild bears [28] and becoming nocturnal in their activity [13], [29]. Studies have shown mixed effects of urbanization on black bear fitness, with positive impacts such as increased litter size [28] and cub survival [30], and negative impacts, such as decreased subadult [31] and adult female survival [25], [30] and overall reduced population growth [30]–[31].

If the fitness benefits associated with urbanization outweigh potential costs, then we can predict that bears should use anthropogenic resources regardless of variations in production of natural food, leading to permanent use of urban areas and irreversible behavioral changes. Alternatively, if bears that forage in urban areas incur fitness costs that are offset only by the temporary scarcity of natural foods, we can predict that resultant behavioral changes relating to bear urbanization will have a strong relationship to seasonal and annual patterns of natural food production, thus resulting in patterns of reversible use of urban areas. The former hypothesis of irreversible behavioral change is often the paradigm for bear management, where it is believed that bears using urban areas become habituated, food-conditioned, and dependent on anthropogenic food sources, leading to “nuisance” behavior and conflicts with humans [22], [32]–[33]. Consequently, bears using urban areas are often removed from the population by lethal or non-lethal (translocation) methods [34], which has the potential to negatively impact local bear populations and can be unpopular with the general public [35]–[36].

Given that by 2050, urbanization is expected to increase by 11% affecting 1.6 million ha across American black bear range in the conterminous U.S. (S. Baruch-Mordo unpublished assessment based on [37]), it is important to understand urban bear ecology to guide management and avoid human-bear conflict and public controversy. In this paper we used detailed GPS location and activity data from a 6-year study of American black bears (*Ursus americanus*) in Aspen, Colorado, USA, to examine the ecology of bears in an urban environment. We assessed bear space use and daily activity patterns and modeled their relationships with bear characteristics and environmental covariates related to seasonal and annual changes in natural food availability. We additionally estimated bear survival and reproductive output to gain insights on potential impacts of urbanization on the local bear population. Overall we assessed the degree of bear urbanization, identified factors that best explained its variation, and asked whether behavioral patterns in use of urban areas were irreversible or fluctuated with natural food availability.

## Materials and Methods

### Ethics Statement

Bear capturing, handling, and monitoring for this research were approved by the Animal Care and Use Committee at Colorado State University (protocols #05-128A and #08-078A). Approval for capturing, handling, and taking samples from bears was granted by Colorado Parks and Wildlife.

### Study Area and Animals

We studied bears in Aspen and the surrounding areas of Pitkin County, located in the central mountains of Colorado (approximately 39.19° longitude and −106.82° latitude; hereafter collectively referred to as Aspen). Elevation in the study area ranges from 2300 to 3150 m. Aspen is situated at the confluence of Maroon, Castle, and Hunter Creeks and the Roaring Fork River, and areas at lower elevation consist of riparian vegetation. With increasing elevation, vegetation changes on south-facing slopes into mountain-shrub community and on north-facing slopes into aspen (Populus tremuloides) and lodgepole (Pinus contorta) forest communities. Mountain-shrub communities primarily consist of the mast producing species (i.e., plants that produce fruits such as acorns and berries) of Gambel oak (Quercus gambelli), serviceberry (Amelancier alnifolia), and chokecherry (Prunus virginiana). Land cover at higher elevations has sparse to no human development and is comprised of Douglas fir (Pseudotsuga menziesii) and spruce (Picea spp.)-subalpine fir (Abies lasiocarpa) coniferous forests, talus slopes, and alpine meadows. The city of Aspen had 6846 residents in 2009 [38], and human housing density varies from 0 – 865 residences per km^2^ (see Space Use section for source and calculations). At its core, Aspen consists of a business district and dense residential areas, and city core is surrounded by less dense residential neighborhoods that are interspersed within the surrounding mountain-shrub and forest communities.

From 2005–2010 we captured 50 bears in the urban environment of Aspen. We defined urban as a land cover characteristic of, and related to, human development [39]. We determined the gender of each bear and used Matson's Laboratory (Milltown, MT, USA) to age bears >1 year old from cementum annuli of their vestigial premolar tooth [40]. We augmented our sample with data from four individuals captured by managers; three were translocated but returned to the study area and one was released near its capture location with aversive conditioning treatments. To avoid potential bias due to capture or management actions, we excluded data collected in the 48 hours following release from capture, or, if translocated, while bears were outside of the study area.

We fitted bears with Lotek© 3300L and 4400M GPS collars that collected a GPS location every 30 minutes from May to September, and every hour in the weeks before and after expected den entry and emergence. Collars also collected activity sensor data that recorded the number of head movements (range 0 – 255) at 5-min intervals throughout collar deployment. We fitted GPS collars with a canvas spacer to allow for drop-off in the event of substantial neck growth, and we programmed mortality sensors to trigger if no activity was logged in a period of several hours. Collars emitting a mortality pulse were investigated in a timely manner to determine whether the bear dropped its collar or died, and for the latter, the cause of death. We monitored bears on a daily basis, and aerially searched for missing individuals outside of the study area every 2–4 weeks. We visited bears during their denning period to replace collar batteries and determine the reproductive status of females.

### Space Use

We estimated home ranges using GPS locations based on positional dilution of precision met the screening criteria: ≤10 for 3D- and ≤5 for 2D-locations [41]–[42]. This resulted in removal on average of 11% (*SE* = 0.75) of locations, and visual examination of the data suggested no effect on overall space use patterns. During hyperphagia bear space use and activity patterns can be altered [43]–[44]; therefore, we stratified analyses by pre-hyperphagia and hyperphagia seasons which were determined based on the fruiting phenology of important food species (Gambel oak, serviceberry, and chokecherry; for approximate phenology dates see USDA, Forest Service, Fire Effects Information System species data <http://www.fs.fed.us/database/feis/>) and the local denning behavior of bears. Pre-hyperphagia included data from the approximate date of den emergence (16 April) to plant fruiting (31 July), and hyperphagia from fruiting to the approximate start date of reduced activity in preparation for denning (15 October). Only bears with data spanning at least 90% of the duration of a given season were included in the analyses.

We estimated seasonal home ranges using the fixed kernel with plug-in bandwidth method [45]–[46]. We implemented analyses using the ks package [47] in program R [48] using the multivariate plug-in function with the Sum of Asymptotic Mean Squared Error pilot option [45]. We defined home range as the polygon resulting from the 95% contour of the utilization distribution, and we generated three response variables to model space use: 1) total home range area (km^2^; Area), 2) amount of overlap (km^2^) between a given seasonal home range and human development (HDoverlap), and 3) mean human density within the home range (HDdensity). We defined human development as areas within a 50-m buffer of human structures, and we used an address layer available for Pitkin County GIS department to generate point density of addresses per 1 km^2^ (range 0 – 865) and calculated the mean density value within the seasonal home range of each bear. Collectively these responses allowed us to evaluate whether bears had smaller home ranges when using urban areas, the degree of overlap with urban areas, and whether this overlap consisted of heavily or sparsely populated areas.

We modeled the three space use response variables as a function of bear age (continuous) and gender, season (pre-hyperphagia and hyperphagia), and the quality of natural forage production (FoodYr). The latter was a qualitative index of good (2005, 2006, 2008, and 2010) and poor (2007 and 2009) food-production years assessed from observed annual yields of the main mast food plants in the study area (i.e., Gambel oak, serviceberry, and chokecherry), and confirmed by local wildlife managers. We note that because mast failure events often occur in response to climatic or disease events (e.g., [49]–[54]), production failures have widespread impacts on multiple plant species. Therefore it was clear from field observations when a poor (or good) natural food year occurred, and such binary index has been used before to qualify mast production (e.g., [55]).

We natural-log transformed space use responses to stabilize the variance and used linear mixed-effects models in nlme package in R [56] where we modeled bears (i.e., used bear id) as a random effect. We ran all possible additive models including an interaction term between season and food year (to allow for different responses during pre-hyperphagia and hyperphagia by food year) for a total of 20 models. We ranked models using AICc, model averaged the parameter estimates, and evaluated fixed effects by examining whether the 95% CI of the model-averaged parameter estimates overlapped zero [57]. We estimated the amount of variability explained by each model as the squared correlation between fitted and observed values.

### Activity Patterns

**Figure 1:**
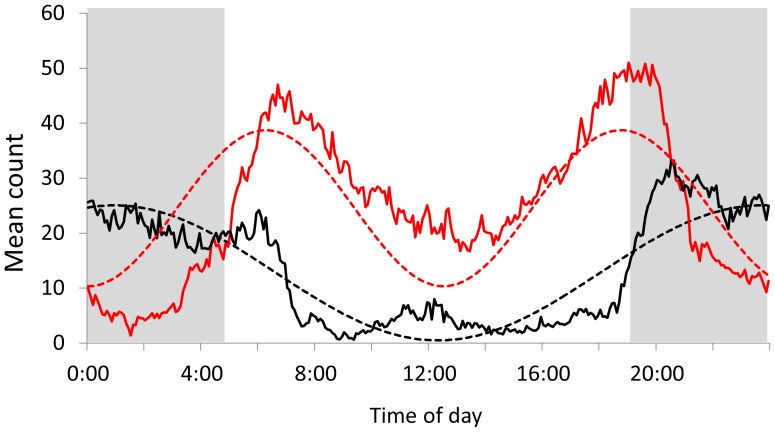
Example sine curves fitted to describe daily activity patterns. Y-axis activity data are summarized for the hyperphagia season in good (red) and poor (black) natural food production years. X-axis data in 0 – 24 hours correspond to a scale of 0 – 2π in radian degrees. Solid lines are the head up-down movements recorded at 5-min intervals and averaged across season, and dashed lines are the fitted sine curves with *b* (number of peaks in 24 hours) and *c* (timing of activity peaks within the 24 hours) parameters equal to 1.92 and 1.57 in a good food year and 1.05 and −1.32 in a poor food year, respectively. Patterns demonstrate crepuscular activity with two peaks (red) and nocturnal activity with a single peak (black).

We developed a new approach to analyze activity patterns and model its changes in relation to individual and environmental covariates. We fitted a sine curve to the mean counts of up-and-down head movements (*y*) collected by GPS collars, and extracted the parameters related to number of peaks (*b*) and x-axis shift (*c*) for the *i_th_* bear, *j_th_* year, and *k_th_* season according to the equation:
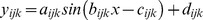
where *a* is amplitude, *x* is time from 0 – 24 hours represented in degrees radian (0 – 2π), and *d* is an offset parameter about the y-axis. We focused analyses on the *b* and *c* parameters because they allowed respective inference on the number and timing of activity bouts within the 24-hour period. For example, nocturnal activity patterns could be described with *b*∼1 and *c*∼−π/2, or one activity bout around midnight (dashed black line; Fig. 1). Conversely, crepuscular activity patterns can be described with *b*∼2 and *c*∼π/2, or a bimodal curve with activity bouts in early morning and late evening (dashed red line; Fig. 1). We used the non-linear least squared (nls) function in R, while bounding *a* and *d* between 0 and 255, *b* between 0 and 5, and *c* between −π/2 and π/2. We used the number of daily peaks and timing of activity bouts as response variables and modeled them as a function of individual and environmental covariates as described above; we used mixed-effects models with individuals as a random effect, ranked models using AICc, evaluated fixed-effects based on 95% CI of model-averaged parameter estimates, and assessed the amount of variability explained by correlating fitted and observed values.

### Survival and Reproduction

We used known-fate models in program MARK [58] to estimate subadult (1–3 years old) and adult (≥4 years old) survival. We created yearly encounter histories with 15 bi-monthly time intervals from April 16 to November 30 and used staggered entry to include bears captured from 2005 – 2010. We assumed survival during the denning period, December 1 – April 15, was 1 [59]–[60]. We censured bears that went missing, dropped their collars, or were removed from the resident population due to translocation. If a bear was recaptured, or if it returned to the study area after translocation, we incorporated it into the analysis. Because some translocated bears returned to our population, we did not consider translocations a mortality event [59], although approximately 40% of the translocated bears (*n* = 13) died while away from Aspen due to control management kills, road kills, harvest, conspecific mortality, or unknown causes. Hence, we acknowledge our survival estimates are likely an overestimate. We modeled effects of gender, age, season (pre-hyperphagia or hyperphagia), food year (good or poor), and season*food year interaction on survival, ranked models using AICc, and model-averaged parameter estimates to calculate unconditional survival estimates [57].

To assess reproductive output, we determined upon capture if females were reproductively active by presence of cubs at capture or at the den (no females showed lactation evidence without having cubs present). We modeled litter size as a function of age of sows and food year during conception using generalized linear models (glm in R, Poisson family) and examined their correlations.

## Results

### Space Use

**Figure 2:**
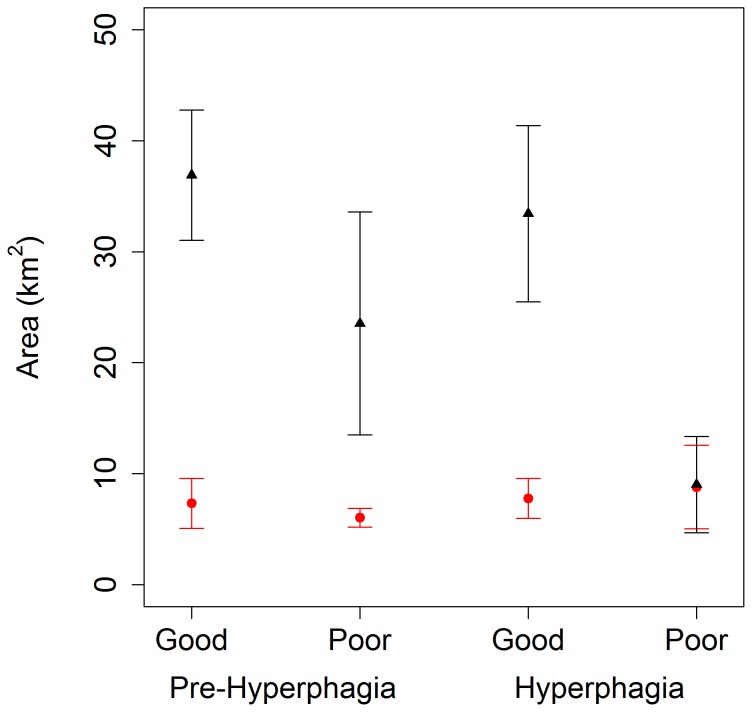
Mean (±1 *SE*) seasonal home range areas (km^2^) for male (black triangle) and female (red circle) bears using Aspen, Colorado, USA. Home ranges were based on GPS locations collected from 2005 – 2010 and calculated as the 95% contour of a utilization distribution estimated using fixed kernel density with a plug-in bandwidth (see methods).

**Figure 3:**
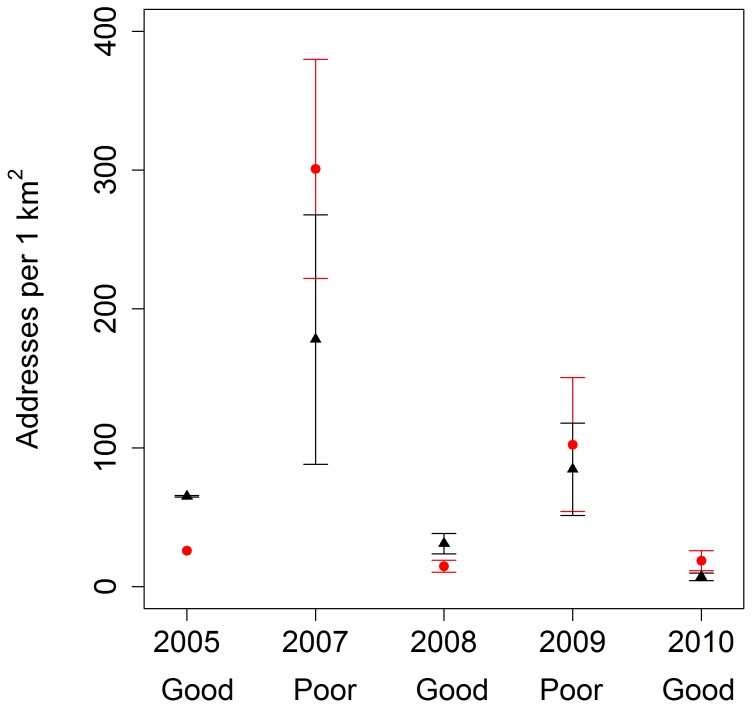
Mean (±1 *SE*) human density in hyperphagia season home ranges of male (black triangle) and female (red circle) bears using Aspen, Colorado, USA from 2005 – 2010. Good or poor year categorization refers to quality of natural food production. Notes: 1) based on our conservative inclusion criteria (i.e., encompassing at least 90% of the season), no data from 2006 met the criteria, and 2) lack of *SE* bars indicate only one sample was available for that year.

**Figure 4:**
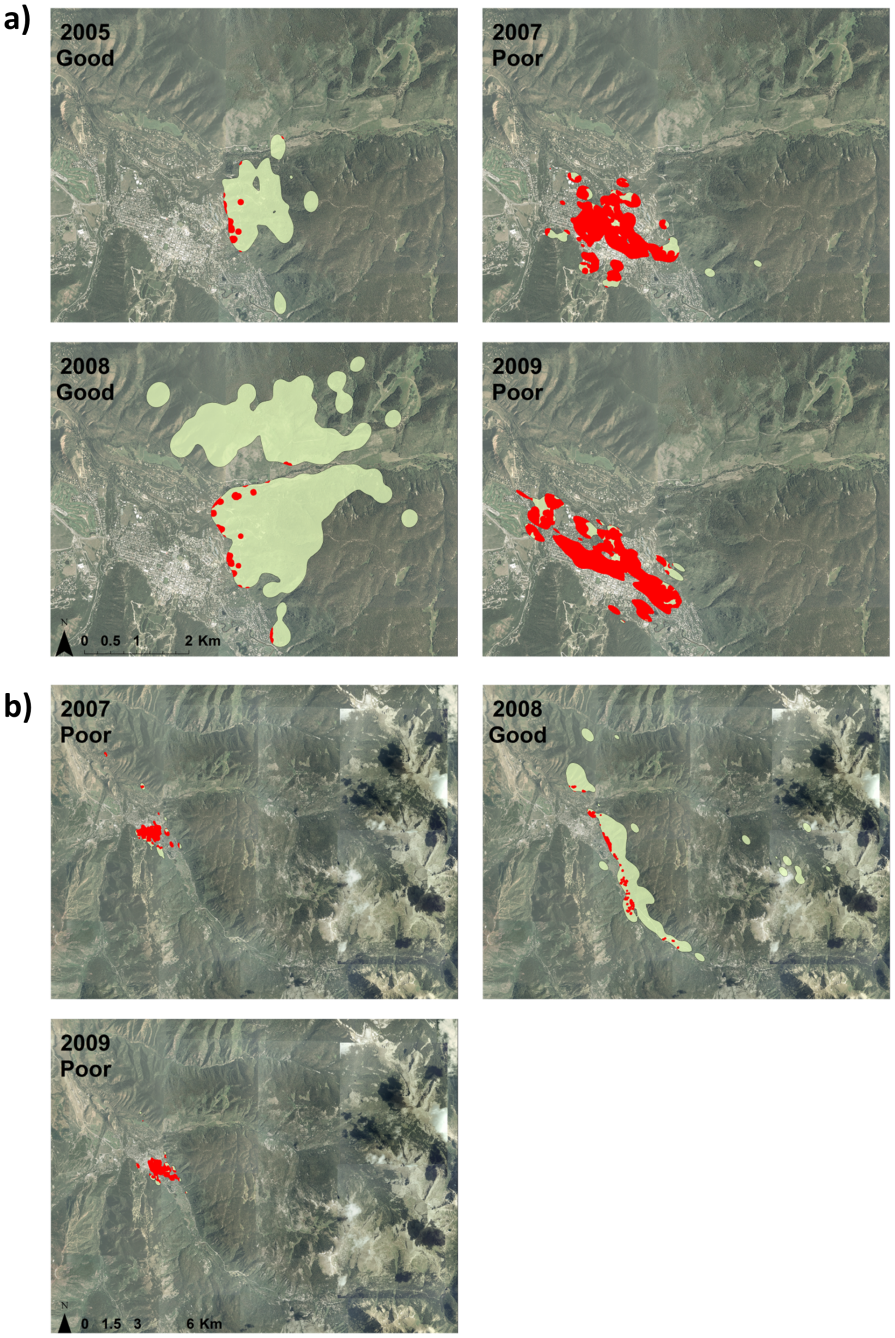
Example shifts in hyperphagia season home ranges (green) and amount of overlap with human development (red) during good and poor natural food production years for bears using Aspen, Colorado, USA. Data were overlaid on an aerial image of Aspen and were presented for a) adult female tracked in 2005, and 2007–2010, and b) an adult male tracked from 2007 – 2009. Patterns demonstrated that in poor natural food years bears had smaller home ranges that were centered on high human density areas in downtown Aspen, but also that bears shifted their home ranges to adjacent wildland areas in subsequent good food years.

We used 57 seasonal home ranges from 23 bears to model space use, where individual bears were monitored from 1–4 years (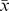  = 1.8, *SE* = 0.2). Models explained on average 60 – 66% of the variability in the data, depending on the response variable (ln(Area): 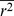  = 0.60, *SE* = 0.01; ln(HDoverlap): 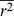  = 0.62, *SE* = 0.01; ln(HDdensity): 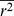  = 0.66, *SE* = 0.02; full model output in Tables S1–S3 in File S1). When modeling ln(Area) as a response, gender appeared in all top models carrying >99% of the weight (Table S1 in File S1), and had a relatively strong effect in each of the models (Table 1). Male home ranges were larger than females, except in the hyperphagia season in poor food years when they were similar to females (Fig. 2). While male home rage area was smallest in hyperphagia of poor food production years, female home range area seemed to stay relatively constant across season and year. Gender and age were always important in explaining variation in the degree of overlap between home range and human development (Table S2 in File S1), where males and younger bears had greater overlap with human development (Table 1). When modeling the mean human housing density within bear home ranges, we found strong support for age and food year effects in each of the models (Table S3 in File S1), with bears having greater mean human density in their home ranges in poor compared to good natural food production years (Fig. 3) and younger bears having greater mean human density in their home ranges compared to other ages (Table 1). During hyperphagia season, bears shifted their home ranges from mostly overlapping high-density downtown areas in poor natural food production years to mostly overlapping adjacent wildland areas in subsequent good natural food years (Fig. 4).

**Table 1:** Model averaged parameter estimates (*SE*) for space use and activity patterns of bears using Aspen, Colorado from 2005 – 2010.

	Space use responses			Activity pattern responses	
Parameter	ln(Area)	ln(HD overlap)	ln(HD density)	*b*	*c*
Intercept	1.470 (0.364)*	1.068 (0.287)*	4.89 (0.581)*	1.335 (0.121)*	−0.511 (0.255)*
Gender (Males)	1.260 (0.343)*	0.553 (0.205)*	0.042 (0.125)	0.001 (0.030)	−0.060 (0.082)
Age	−0.006 (0.009)	−0.034 (0.016)*	−0.080 (0.038)*	0.000 (0.003)	−0.008 (0.009)
Season (Pre-Hyperphagia)	0.270 (0.229)	0.035 (0.056)	−0.175 (0.182)	0.414 (0.141)*	1.916 (0.421)*
Food Year (Good)	0.369 (0.221)	−0.242 (0.124)	−1.82 (0.312)*	0.129 (0.093)	1.663 (0.278)*
Season*Food Year	−0.224 (0.205)	0.002 (0.016)	0.082 (0.107)	−0.026 (0.047)	−1.595 (0.487)*

Responses were modeled as a function of bear characteristics (age, gender), season (pre-hyperphagia, hyperphagia), and natural food production year (good, poor). Space use responses (log-transformed) were estimated from GPS locational data using a fixed kernel home range methods and include 1) total home range area in km^2^, ln(Area), 2) amount of home range overlap with human development in km^2^, ln(HD overlap), and 3) mean human density within the home range, ln(HD density). Activity pattern responses were estimated by fitting a sine curve to the daily mean counts of up-and-down head movements collected by GPS collars and included the number of activity peaks (*b*) and timing of activity bouts (c) within a 24-hour period.

Indicates 95% *CI* did not overlap zero.

### Activity Patterns

**Figure 5:**
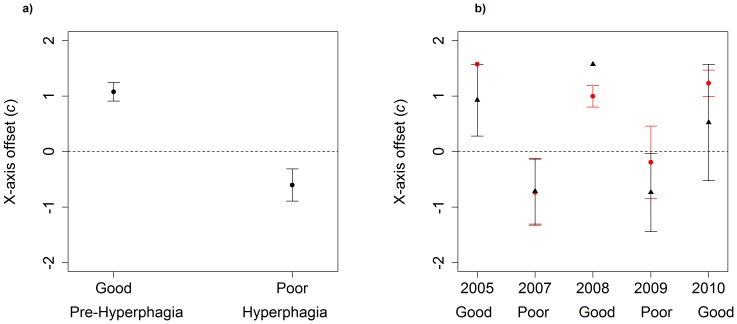
Mean (±1 *SE*) x-axis offset shape parameter (*c*) by a) season and year, and b) gender (males black triangle, females red circle) and year for the hyperphagia season, for bears using Aspen, Colorado, USA from 2005 – 2010. Good or poor year categorization refers to quality of natural food production. The x-axis offset shape parameter was extracted by fitting a sine curve to seasonal activity data of bears; negative values tending towards −π/2 indicate nocturnal activity and positive values tending towards π/2 indicate diurnal activity. Notes: 1) based on our conservative inclusion criteria (i.e., encompassing at least 90% of the season), no data from 2006 met the criteria, and 2) lack of *SE* bars indicate only one sample was available for that year.

**Figure 6:**
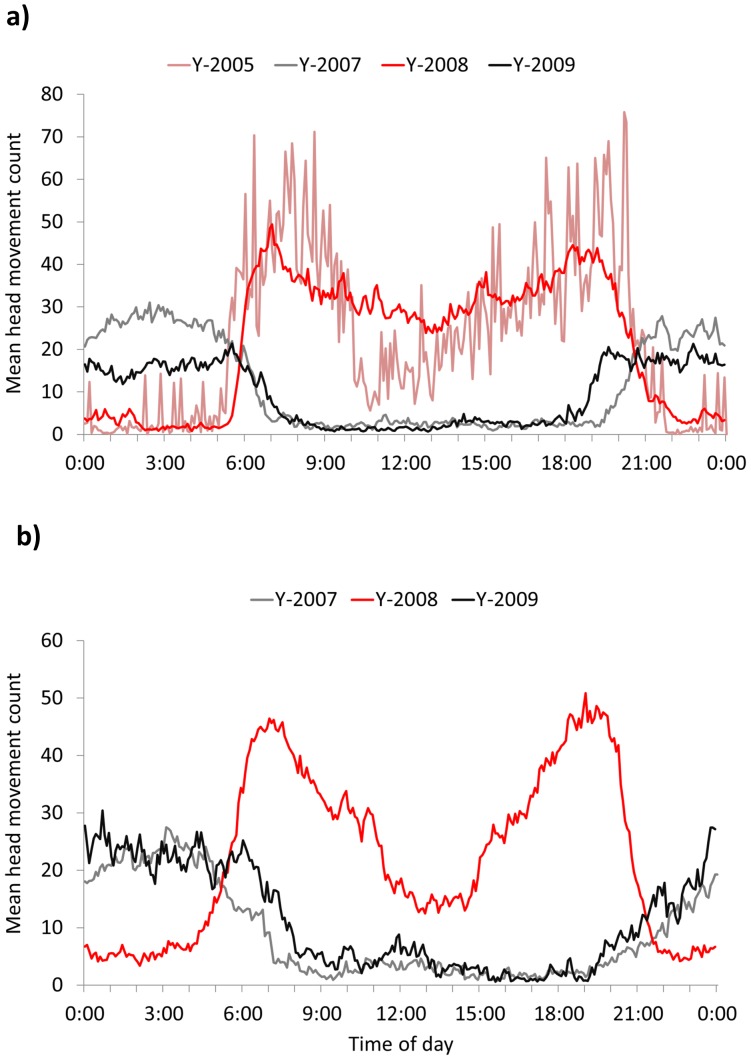
Example shifts in activity patterns in good (red and pink) and poor (black and grey) natural food years for bears using Aspen, Colorado, USA. Head movement data were collected from sensors in GPS collars and averaged for the hyperphagia season for a) adult female tracked in 2005, and 2007 – 2010 and b) adult male tracked from 2007 – 2009. Patterns demonstrate that in poor natural food years bears became more nocturnal and unimodal in their activity, but also became diurnal and bimodal in subsequent good food years.

We fitted 61 seasonal activity curves for 25 bears to extract the number of activity peaks (*b*) and their timing in the 24-hour period (*c*) and to model activity patterns. Individual bears were monitored from 1–4 years (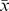  = 1.8, *SE* = 0.19). Models explained up to 52% of the variability in the data and on average, explained more variability in *c* (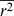  = 0.29, *SE* = 0.07) compared to *b* (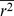  = 0.14, *SE* = 0.03; full model output in Tables S4–S5 in File S2). Season was the only important predictor of number of peaks in activity (*b*; Table S4 in File S2), where modality increased during pre-hyperphagia (Table 1). Season, food year, and season*food year interaction were important predictors of timing of daily activity (*c*; Table S5 in File S2). Unconditional parameter estimates for season and food year were positive (Table 1), indicating that bears were more active during daylight hours during pre-hyperphagia and in good food production years. Parameter estimate for season*food was negative (Table 1), with both females and males becoming more nocturnal during hyperphagia in poor natural food production years (Fig. 5). Similar to space use results, bears shifted their activity patterns to diurnal and bimodal in subsequent good food years (Fig. 6).

### Survival and Reproduction

We recorded 6 mortalities from harvest (*n* = 1), conflict management (*n* = 4), and unknown (*n* = 1) causes. We included 63 yearly encounter histories for 39 bears in the known-fate models, and we censured 27 capture histories due to dropped collars or translocations. Survival was lower in poor food years for all gender and age combinations, where model-averaged estimates ranged from 0.675 (*SE* = 0.158) for subadult males to 0.718 (*SE* = 0.117) for adult females (Table 2). Food year was an important factor explaining variability in survival and appeared in all top models carrying >98% of the weight (full model output in Table S6 in File S3).

**Table 2:** Unconditional annual survival estimates (*SE*) for bears using Aspen, Colorado from 2005 – 2010.

	Males	Females
	Poor food year	
Subadults	0.675 (0.154)	0.707 (0.141)
Adults	0.684 (0.137)	0.718 (0.117)
	Good food year	
Subadults	0.998 (0.020)	0.998 (0.017)
Adults	0.998 (0.019)	0.998 (0.016)

Gender-specific subadult (1–3 years old) and adult (≥4 years old) survival was calculated for poor and good natural food production years using known fate models in program MARK.

We documented 19 litters totaling 42 cubs that were produced by 13 females of ages 3 – 20 years. Litter size varied from 1 – 3 cubs (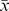  = 2.21, *SE* = 0.18), and all litters with 1 cub were born to females ≤5 years old. There was no relationship between mean litter size based on conception in good (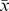  = 2.4, *SE* = 0.16) versus poor (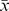  = 2.0, *SE* = 0.20) years, nor between litter size and female's age at conception (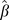  = 0.02, *SE* = 0.03) or food year (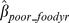  = −0.14, *SE* = 0.37).

## Discussion

We evaluated the degree of bear urbanization in Aspen, Colorado, USA, and explored the factors that best explained its variations. Bears demonstrated temporal fluctuations in space use and activity-pattern behaviors that were strongly dependent on the availability of natural food resources. During poor natural food years bears used dense human development areas and were more active at night, but they also demonstrated behavioral plasticity where in subsequent good natural food years they reversed their behavior to daytime foraging away from urban areas. When bears used urban areas in poor food years they had lower survival compared to good food years with most mortality being human-caused. Collectively our data suggests that in some systems bear use of urban areas can be reversible and fluctuate with the availability of natural food, and that such patterns can negatively impact bear survival.

When bears used urban areas in poor natural food years patterns of space use (smaller home ranges) and activity (nocturnal) were similar to those reported for black bears [13], [29] and other species, e.g., Alpine cough (*Pyrrhocorax graculus*) [17], Florida Key deer (*Odocoileus virginianus clavium*) [18], and Northern Cardinal (*Cardinalis cardinalis*) [14]. Additionally, the changes in space use behavior in response to mast failures were similar to those of wild Asiatic black bears (*Ursus thibetanus*), which shifted their home ranges depending on whether mast production was poor or good [55]. However the patterns of fluctuating use of urban areas observed in this study contradicted results from a detailed and comprehensive study of black bear ecology in urban areas of Lake Tahoe, Nevada USA, where bears appeared to have an irreversible dependency on human foods [13]. We hypothesize that a reason for the difference is the landscape context of the two studies. Lake Tahoe is surrounded by large desert basins that are marginal habitats for bears [61], whereas habitats surrounding Aspen are considered one of the most productive in Colorado [62]. Consequently Aspen bears have good natural resources to shift back to in good food years, but such resources may not be available to Lake Tahoe bears. Therefore, the landscape matrix in which an urban area is situated is likely to affect whether individuals become irreversibly urban and should be considered when managing local wildlife populations.

Several authors suggested that urban areas can serve as refuges for wildlife in times of low natural food production providing a safeguard against mortality, reproduction failure, and overall population decline [32], [63]. For example, in India urban Hanuman langur (Seemnopithecus entellus) populations avoided massive die offs during La Niña drought events by feeding on anthropogenic foods [63]; in Poland black-billed magpies (Pica pica) with access to anthropogenic foods had lower nest failure during inclement weather [64]; and in California USA, urban kit foxes (Vulpes macrotis) were in better physiological condition than their rural counterparts during a 2-year drought event [9]. The fact that black bears in our study increased their degree of urbanization during poor food years may at first glance lend support for a refuge hypothesis. However if survival is reduced due to increased human-caused mortality resulting from management of human-bear conflict, then urban areas may not serve as refuges for bears but instead can present ecological and even evolutionary traps [65]–[66].

Adult female survival of black bears is generally high and is believed to influence population growth more than recruitment [67]–[68], and evidence suggests that adult female survival is similar between good and poor natural food years ([69], [70]; but see [71]). In our study, survival of adult female bears in good food years (1.0, Table 2) was comparable to those of wildland bears in south-central Colorado (range 0.92 – 1.0) [62], and in Rocky Mountain National Park in north-central Colorado (1.0, *SE* = 0.0) [72]. Adult female survival was lower for our study bears in poor food years (0.76), but estimates were similar to female bears occupying residential areas in Florida, USA (0.776, *SE* = 0.074) [30] and all management bears (i.e., male and female bears defined as problem bears) in Alberta, Canada (0.66, *SE* = 0.113) [59]. Although we did not concurrently monitor wildland bear populations, the fact that 1) survival in good years was comparable to published estimates of survival from wildland populations, 2) adult survival is a less variable demographic parameter with some studies showing that it is similar in poor natural food years, and 3) population growth is sensitive to changes in adult female survival, suggests that low survival rates of females in Aspen in poor natural food years may be creating a population sink rather than a refuge.

During poor natural food years mortality of bears increases and is largely human-caused resulting from conflicts near human development [25], [27], [73]. Because urban areas can attract bears in poor food years, a time when the population growth may already be stressed, removal of bears that use the urban environment could negatively affect the population locally or regionally depending on the attraction distance of urban areas. Under current predictions urbanization is expected to continue to increase across American black bear range [37] and natural food failures, which are often linked to weather events such as late spring frost and drought, may increase in frequency given global climate change [74], [75]. These combined trends can further increase bear use of urban areas and human-bear conflict. Certainly to minimize safety risks to people, removal of some bears will be required; however, increased tolerance will be important when management goals are to sustain local bear populations. Furthermore, managers can limit recreational harvest to reduce overall mortality during years of poor natural food production which can be predicted by early season (or even previous year) weather events (e.g., [52]–[54], [76], [77]). Finally, managers can focus on reducing the availability of anthropogenic resources that attract bears to urban areas, e.g., garbage and fruit trees, thereby providing long-term solutions for the coexistence of people and bears [32], [34], [78]–[80].

## Supporting Information

File S1 **Tables S1–S3.** Full model set and model averaged parameter results for space use.
(DOC)

File S2 **Tables S4–S5.** Full model set and model averaged parameter results for activity patterns.
(DOC)

File S3 **Table S6.** Full model set and results for known-fate survival.
(DOC)
