# Supplementary material for: Stochasticity in Natural Forage Production Affects Use of Urban Areas by Black Bears: Implications to Management of Human-Bear Conflicts
Source: PLoS One. 2014 Jan 8;9(1):e85122. doi: 10.1371/journal.pone.0085122 (PMC3885671; doi:10.1371/journal.pone.0085122)
Supplement: File S1 — Tables S1–S3. Full model set and model averaged parameter results for space use. (DOC) [file pone.0085122.s001.doc]

**Tables S1 – S3.** Full model set results for modeling of space use of urban black bears in Aspen, Colorado, USA from 2005 - 2010. Response variables include natural-log transformed seasonal home range area (km^2^; ln(Area); Table S1), amount of overlap of home range with human development (km^2^; ln(HD overlap); Table S2), and mean human density within the home range (number of addresses/km^2^; ln(HD density); Table S3). Home ranges were calculated as the 95% contour of a utilization distribution estimated using kernel density with a plug-in bandwidth approach. Seasons were defined as pre-hyperphagia (April 15 – July 31) and hyperphagia (August 1 – October 15), and natural food production years (FoodYr) were defined as poor or good based on qualitative assessment of yield of important mast producing plants in the study area.

**Table S1.** Model set results, response = ln(Area).

| Model | *r^2*^* | *k*^†^ | AICc | ∆AICc | *w* |
| --- | --- | --- | --- | --- | --- |
| Gender + FoodYr | 0.57 | 5 | 158.86 | 0.00 | 0.23 |
| Gender + Season + FoodYr + Season*FoodYr | 0.64 | 7 | 159.32 | 0.46 | 0.18 |
| Gender | 0.54 | 4 | 159.89 | 1.03 | 0.13 |
| Gender + Season + FoodYr | 0.60 | 6 | 160.47 | 1.61 | 0.10 |
| Gender + Age + FoodYr | 0.57 | 6 | 160.85 | 1.98 | 0.08 |
| Gender + Season | 0.58 | 5 | 161.17 | 2.31 | 0.07 |
| Gender + Age + Season + FoodYr + Season*FoodYr | 0.64 | 8 | 161.29 | 2.43 | 0.07 |
| Gender + Age | 0.54 | 5 | 161.59 | 2.72 | 0.06 |
| Gender + Age + Season + FoodYr | 0.60 | 7 | 162.36 | 3.50 | 0.04 |
| Gender + Age + Season | 0.58 | 6 | 162.73 | 3.87 | 0.03 |
| Intercept only | 0.59 | 3 | 168.74 | 9.88 | 0.00 |
| FoodYr | 0.61 | 4 | 169.33 | 10.47 | 0.00 |
| Season | 0.62 | 4 | 170.38 | 11.52 | 0.00 |
| Age | 0.58 | 4 | 170.43 | 11.56 | 0.00 |
| Season + FoodYr + Season*FoodYr | 0.66 | 6 | 170.45 | 11.59 | 0.00 |
| Season + FoodYr | 0.63 | 5 | 171.18 | 12.32 | 0.00 |
| Age + FoodYr | 0.60 | 5 | 171.22 | 12.36 | 0.00 |
| Age + Season | 0.61 | 5 | 172.02 | 13.16 | 0.00 |
| Age + Season + FoodYr + Season*FoodYr | 0.66 | 7 | 172.35 | 13.49 | 0.00 |
| Age + Season + FoodYr | 0.63 | 6 | 173.05 | 14.18 | 0.00 |

* Amount of variability explained by each model (*r^2^*) was calculated as the squared correlation between fitted and observed values.

† Number of parameters (*k*) was calculated as the number of fixed-effects parameters plus three for the intercept, random effects, and overall variance.

**Table S2.** Model set results, response = ln(HD overlap).

| Model | *r^2^* | *k* | AICc | ∆AICc | *W* |
| --- | --- | --- | --- | --- | --- |
| Gender + Age + FoodYr | 0.63 | 6 | 101.13 | 0.00 | 0.39 |
| Gender + Age + Season + FoodYr | 0.65 | 7 | 102.82 | 1.69 | 0.17 |
| Gender + Age | 0.60 | 5 | 103.56 | 2.42 | 0.11 |
| Gender + FoodYr | 0.58 | 5 | 104.14 | 3.01 | 0.09 |
| Gender + Age + Season + FoodYr + Season*FoodYr | 0.65 | 8 | 105.53 | 4.39 | 0.04 |
| Gender | 0.54 | 4 | 105.56 | 4.43 | 0.04 |
| Gender + Age + Season | 0.60 | 6 | 105.59 | 4.45 | 0.04 |
| Age + FoodYr | 0.64 | 5 | 105.98 | 4.85 | 0.03 |
| Gender + Season + FoodYr | 0.60 | 6 | 106.30 | 5.16 | 0.03 |
| Gender + Season | 0.55 | 5 | 107.82 | 6.69 | 0.01 |
| Age + Season + FoodYr | 0.65 | 6 | 107.99 | 6.86 | 0.01 |
| Gender + Season + FoodYr + Season*FoodYr | 0.60 | 7 | 108.90 | 7.76 | 0.01 |
| FoodYr | 0.63 | 4 | 108.92 | 7.79 | 0.01 |
| Age | 0.62 | 4 | 109.41 | 8.27 | 0.01 |
| Age + Season + FoodYr + Season*FoodYr | 0.66 | 7 | 110.57 | 9.43 | 0.00 |
| Season + FoodYr | 0.64 | 5 | 111.18 | 10.05 | 0.00 |
| Intercept only | 0.60 | 3 | 111.45 | 10.32 | 0.00 |
| Age + Season | 0.62 | 5 | 111.66 | 10.53 | 0.00 |
| Season + FoodYr + Season*FoodYr | 0.64 | 6 | 113.64 | 12.51 | 0.00 |
| Season | 0.60 | 4 | 113.76 | 12.62 | 0.00 |

* Amount of variability explained by each model (*r^2^*) was calculated as the squared correlation between fitted and observed values.

† Number of parameters (*k*) was calculated as the number of fixed-effects parameters plus three for the intercept, random effects, and overall variance.

**Table S3.** Model set results, response = ln(HD density).

| Model | *r^2^* | *k* | AICc | ∆AICc | *w* |
| --- | --- | --- | --- | --- | --- |
| Age + FoodYr | 0.75 | 5 | 189.50 | 0.00 | 0.37 |
| Age + Season + FoodYr | 0.76 | 6 | 191.02 | 1.52 | 0.17 |
| Gender + Age + FoodYr | 0.75 | 6 | 191.84 | 2.34 | 0.11 |
| Age + Season + FoodYr + Season*FoodYr | 0.77 | 7 | 192.49 | 3.00 | 0.08 |
| FoodYr | 0.69 | 4 | 192.56 | 3.07 | 0.08 |
| Gender + Age + Season + FoodYr | 0.76 | 7 | 193.53 | 4.03 | 0.05 |
| Season + FoodYr | 0.72 | 5 | 193.59 | 4.09 | 0.05 |
| Gender + FoodYr | 0.69 | 5 | 194.73 | 5.23 | 0.03 |
| Gender + Age + Season + FoodYr + Season*FoodYr | 0.77 | 8 | 195.12 | 5.63 | 0.02 |
| Season + FoodYr + Season*FoodYr | 0.74 | 6 | 195.12 | 5.63 | 0.02 |
| Gender + Season + FoodYr | 0.72 | 6 | 195.96 | 6.46 | 0.01 |
| Gender + Season + FoodYr + Season*FoodYr | 0.74 | 7 | 197.61 | 8.11 | 0.01 |
| Intercept only | 0.51 | 3 | 216.29 | 26.79 | 0.00 |
| Age | 0.56 | 4 | 216.68 | 27.19 | 0.00 |
| Season | 0.54 | 4 | 216.93 | 27.43 | 0.00 |
| Gender | 0.49 | 4 | 217.59 | 28.09 | 0.00 |
| Age + Season | 0.58 | 5 | 217.62 | 28.13 | 0.00 |
| Gender + Age | 0.55 | 5 | 218.21 | 28.72 | 0.00 |
| Gender + Season | 0.53 | 5 | 218.63 | 29.13 | 0.00 |
| Gender + Age + Season | 0.57 | 6 | 219.50 | 30.00 | 0.00 |

* Amount of variability explained by each model (*r^2^*) was calculated as the squared correlation between fitted and observed values.

† Number of parameters (*k*) was calculated as the number of fixed-effects parameters plus three for the intercept, random effects, and overall variance.
