# Supplementary material for: Stochasticity in Natural Forage Production Affects Use of Urban Areas by Black Bears: Implications to Management of Human-Bear Conflicts
Source: PLoS One. 2014 Jan 8;9(1):e85122. doi: 10.1371/journal.pone.0085122 (PMC3885671; doi:10.1371/journal.pone.0085122)
Supplement: File S2 — Tables S4–S5. Full model set and model averaged parameter results for activity patterns. (DOC) [file pone.0085122.s002.doc]

**Tables S4 – S5.** Full model set results for modeling of activity patterns of urban black bears in Aspen, Colorado, USA from 2005 - 2010. Response variables include number of peaks (*b*; Table S4) and timing (*c*; Table S5) of daily activity patterns, and were extracted by fitting a sine curve to mean count of up-down head movements for each season, year, and bear. Seasons were defined as pre-hyperphagia (April 15 – July 31) and hyperphagia (August 1 – October 15), and natural food production years (FoodYr) were defined as poor or good based on qualitative assessment of yield of important mast producing plants in the study area.

**Table S4.** Model set results, response = *b* number of activity peaks within a 24-hour period.

| Model | *r^2*^* | *k*^†^ | AICc | ∆AICc | *w* |
| --- | --- | --- | --- | --- | --- |
| Season + FoodYr | 0.20 | 5 | 84.26 | 0.00 | 0.27 |
| Season | 0.16 | 4 | 84.60 | 0.35 | 0.22 |
| Season + FoodYr + Season*FoodYr | 0.21 | 6 | 86.30 | 2.04 | 0.10 |
| Gender + Season + FoodYr | 0.20 | 6 | 86.70 | 2.44 | 0.08 |
| Age + Season + FoodYr | 0.20 | 6 | 86.71 | 2.45 | 0.08 |
| Gender + Season | 0.16 | 5 | 86.96 | 2.71 | 0.07 |
| Age + Season | 0.16 | 5 | 86.97 | 2.71 | 0.07 |
| Age + Season + FoodYr + Season*FoodYr | 0.21 | 7 | 88.83 | 4.57 | 0.03 |
| Gender + Season + FoodYr + Season*FoodYr | 0.21 | 7 | 88.83 | 4.58 | 0.03 |
| Gender + Age + Season + FoodYr | 0.20 | 7 | 89.25 | 5.00 | 0.02 |
| Gender + Age + Season | 0.16 | 6 | 89.41 | 5.16 | 0.02 |
| Gender + Age + Season + FoodYr + Season*FoodYr | 0.21 | 8 | 91.47 | 7.21 | 0.01 |
| FoodYr | 0.06 | 4 | 91.74 | 7.48 | 0.01 |
| Intercept only | 0.18 | 3 | 93.21 | 8.95 | 0.00 |
| Age + FoodYr | 0.06 | 5 | 93.87 | 9.61 | 0.00 |
| Gender + FoodYr | 0.06 | 5 | 94.07 | 9.81 | 0.00 |
| Gender | 0.00 | 4 | 95.19 | 10.94 | 0.00 |
| Age | 0.00 | 4 | 95.20 | 10.94 | 0.00 |
| Gender + Age + FoodYr | 0.06 | 6 | 96.32 | 12.06 | 0.00 |
| Gender + Age | 0.01 | 5 | 97.38 | 13.12 | 0.00 |

* Amount of variability explained by each model (*r^2^*) was calculated as the squared correlation between fitted and observed values.

† Number of parameters (*k*) was calculated as the number of fixed-effects parameters plus three for the intercept, random effects, and overall variance.

**Table S5.** Model set results, response = *c* timing of activity within a 24-hour period.

| Model | *r^2^* | *k* | AICc | ∆AICc | *w* |
| --- | --- | --- | --- | --- | --- |
| Season + FoodYr + Season*FoodYr | 0.50 | 6 | 162.85 | 0.00 | 0.47 |
| Age + Season + FoodYr + Season*FoodYr | 0.51 | 7 | 164.31 | 1.47 | 0.23 |
| Gender + Season + FoodYr + Season*FoodYr | 0.51 | 7 | 164.75 | 1.91 | 0.18 |
| Gender + Age + Season + FoodYr + Season*FoodYr | 0.52 | 8 | 165.85 | 3.00 | 0.10 |
| Season + FoodYr | 0.41 | 5 | 170.85 | 8.00 | 0.01 |
| Gender + Season + FoodYr | 0.41 | 6 | 172.69 | 9.84 | 0.00 |
| Age + Season + FoodYr | 0.41 | 6 | 172.82 | 9.97 | 0.00 |
| Gender + Age + Season + FoodYr | 0.42 | 7 | 174.45 | 11.60 | 0.00 |
| FoodYr | 0.29 | 4 | 179.19 | 16.34 | 0.00 |
| Gender + FoodYr | 0.31 | 5 | 180.38 | 17.53 | 0.00 |
| Age + FoodYr | 0.29 | 5 | 181.56 | 18.72 | 0.00 |
| Gender + Age + FoodYr | 0.31 | 6 | 182.77 | 19.93 | 0.00 |
| Season | 0.17 | 4 | 189.12 | 26.28 | 0.00 |
| Gender + Season | 0.19 | 5 | 189.70 | 26.86 | 0.00 |
| Age + Season | 0.17 | 5 | 191.18 | 28.34 | 0.00 |
| Gender + Age + Season | 0.20 | 6 | 191.40 | 28.56 | 0.00 |
| Gender | 0.04 | 4 | 197.48 | 34.64 | 0.00 |
| Intercept only | 0.23 | 3 | 197.96 | 35.11 | 0.00 |
| Gender + Age | 0.05 | 5 | 199.80 | 36.95 | 0.00 |
| Age | 0.00 | 4 | 200.23 | 37.39 | 0.00 |

* Amount of variability explained by each model (*r^2^*) was calculated as the squared correlation between fitted and observed values.

† Number of parameters (*k*) was calculated as the number of fixed-effects parameters plus three for the intercept, random effects, and overall variance.
