# Supplementary material for: Stochasticity in Natural Forage Production Affects Use of Urban Areas by Black Bears: Implications to Management of Human-Bear Conflicts
Source: PLoS One. 2014 Jan 8;9(1):e85122. doi: 10.1371/journal.pone.0085122 (PMC3885671; doi:10.1371/journal.pone.0085122)
Supplement: File S3 — Table S6. Full model set and results for known-fate survival. (DOC) [file pone.0085122.s003.doc]

**Table S6.** Full model output from program MARK for known-fate models estimating survival of urban black bears in Aspen, Colorado, USA from 2005 - 2010. Seasons were defined as pre-hyperphagia (April 15 – July 31) and hyperphagia (August 1 – October 15), and natural food production years (FoodYr) were defined as poor or good based on qualitative assessment of yield of important mast producing plants in the study area.

| Model | *k** | AICc | ∆AICc | *w* | Model Likelihood |
| --- | --- | --- | --- | --- | --- |
| Season + FoodYr | 4 | 62.44 | 0.00 | 0.24 | 1.00 |
| FoodYr | 3 | 62.99 | 0.55 | 0.18 | 0.76 |
| Gender + Season + FoodYr | 5 | 64.15 | 1.72 | 0.10 | 0.42 |
| Age + Season + FoodYr | 5 | 64.41 | 1.97 | 0.09 | 0.37 |
| Season + FoodYr + Season*FoodYr | 5 | 64.47 | 2.03 | 0.09 | 0.36 |
| Gender + FoodYr | 4 | 64.80 | 2.37 | 0.07 | 0.31 |
| Age + FoodYr | 4 | 65.00 | 2.57 | 0.07 | 0.28 |
| Gender + Age + Season + FoodYr | 6 | 66.16 | 3.72 | 0.04 | 0.16 |
| Gender + Season + FoodYr + Season*FoodYr | 6 | 66.20 | 3.76 | 0.04 | 0.15 |
| Age + Season + FoodYr + Season*FoodYr | 6 | 66.45 | 4.02 | 0.03 | 0.13 |
| Gender + Age + FoodYr | 5 | 66.84 | 4.40 | 0.03 | 0.11 |
| Gender + Age + Season + FoodYr + Season*FoodYr | 7 | 68.21 | 5.77 | 0.01 | 0.06 |
| Season | 3 | 71.08 | 8.65 | 0.00 | 0.01 |
| Intercept only | 2 | 71.24 | 8.80 | 0.00 | 0.01 |
| Gender + Season | 4 | 72.65 | 10.21 | 0.00 | 0.01 |
| Gender + Age + Season | 4 | 72.65 | 10.21 | 0.00 | 0.01 |
| Gender | 3 | 72.89 | 10.45 | 0.00 | 0.01 |
| Age + Season | 4 | 73.10 | 10.66 | 0.00 | 0.00 |
| Age | 3 | 73.26 | 10.82 | 0.00 | 0.00 |
| Gender + Age | 4 | 74.91 | 12.48 | 0.00 | 0.00 |

* Number of parameters (*k*) was calculated as the number of parameters plus two for the intercept and overall variance.
